# Tick-borne pathogens and the vector potential of ticks in China

**DOI:** 10.1186/s13071-014-0628-x

**Published:** 2015-01-14

**Authors:** Zhijun Yu, Hui Wang, Tianhong Wang, Wenying Sun, Xiaolong Yang, Jingze Liu

**Affiliations:** Key Laboratory of Animal Physiology, Biochemistry and Molecular Biology of Hebei Province, College of Life Sciences, Hebei Normal University, Shijiazhuang, 050024 China

**Keywords:** Ticks, Tick-borne pathogens, Vector potential, China

## Abstract

Ticks, as obligate blood-sucking ectoparasites, attack a broad range of vertebrates and transmit a great diversity of pathogenic microorganisms. They are considered second only to mosquitoes as vectors of human disease, and the most important vector of pathogens of domestic and wild animals. Of the 117 described species in the Chinese tick fauna, 60 are known to transmit one or more diseases: 36 species isolated within China and 24 species isolated outside China. Moreover, 38 of these species carry multiple pathogens, indicating the potentially vast role of these vectors in transmitting pathogens. Spotted fever is the most common tick-borne disease, and is carried by at least 27 tick species, with Lyme disease and human granulocytic anaplasmosis ranked as the second and third most widespread tick-borne diseases, carried by 13 and 10 species, respectively. Such knowledge provides us with clues for the identification of tick-associated pathogens and suggests ideas for the control of tick-borne diseases in China. However, the numbers of tick-associated pathogens and tick-borne diseases in China are probably underestimated because of the complex distribution and great diversity of tick species in this country.

## Review

Ticks, as obligate blood-sucking ectoparasites, attack a broad range of vertebrates, including humans, and they are considered second only to mosquitoes as vectors of human disease, and the most important vector of pathogens of domestic and wild animals [1]. They transmit a variety of pathogens of medical and veterinary interest, including viruses, bacteria, rickettsiae, helminthes, and protozoans, all of which are able to cause damage to livestock production and human health. The global threat of tick-borne diseases is increasing, with new pathogens identified continuously [2]. There are an estimated 899 species of ticks belonging to three families: Argasidae, Ixodidae, and Nuttalliellidae (represented by a monotypic species restricted to South Africa) [3].

In China, 117 species of the following genera have been identified: *Argas* (seven species), *Carios* (four species), and *Ornithodoros* (two species) in the family Argasidae; and *Amblyomma* (eight species), *Anomalohimalaya* (two species), *Dermacentor* (twelve species), *Haemaphysalis* (forty four species), *Hyalomma* (six species), *Ixodes* (twenty four species), and *Rhipicephalus* (eight species) in the family Ixodidae [4]. Some of these species carry or transmit one or more infectious pathogens, resulting in severe zoonotic diseases. The most commonly observed human tick-borne diseases in China are reportedly Lyme disease, tick-borne encephalitis, Crimean-Congo hemorrhagic fever, Q fever, tularemia, and North-Asia tick-borne spotted fever [5]. Epidemiologically important tick-borne diseases, such as Human Granulocytic Anaplasmosis (HGA) and severe Fever with Thrombocytopenia Syndrome (FLTS), have also emerged in recent years. The characterization of a new bunyavirus (associated with fever, thrombocytopenia, and leukopenia syndrome) in 2010 has prompted greater attention to ticks and tick-borne diseases throughout China. However, tick-associated pathogens and diseases are still underestimated because of the complex distribution and the large diversity of tick species in China.

Although the rapid development of molecular techniques has greatly advanced the identification of emerging tick pathogens, continuous research is required to fully comprehend the diversity of tick-borne pathogens and to completely identify the vector roles of ticks in China. In this study, with regard to the Chinese tick fauna, we reviewed the tick-associated pathogenic microorganisms that have been identified world-wide, and evaluated the potential roles of the ticks as vectors throughout China. This will extend the identification of tick-associated pathogens and suggest better strategies for the control of tick-borne diseases in China.

### Role of argasid ticks as vectors in China and their associated tick-borne pathogens

In China, there are 13 species of argasid ticks, belonging to three genera: *Argas* (seven species), *Carios* (four species), and *Ornithodoros* (two species) [4]. The majority of these are nidicolous, usually residing in the burrows, caves, or nests of their hosts. Among all the argasids found in China, four *Argas* species, two *Carios* species, and two *Ornithodoros* species are competent to transmit or cause human disease (Table 1) [6-17]. Among these eight tick species, four (*A. japonicas*, *A. persicus*, *O. tartakovskyi*, and *O. tholozani*) have been confirmed as causing host illnesses in China. A case of human dermatitis was recorded in 1986 after a bite by *A. japonicas*, but no pathogen has been identified from this tick species in China [6]. The tick *A. persicus* mainly infests poultry and carries the most diverse array of pathogens in the family Agarsidae, including *Borrelia anserine*, Kyasanur Forest disease virus, and *Wolbachia persica* n. sp. However, only *B. anserine*, known to cause avian spirochetosis, has been confirmed in China [7]. Lake Clarendon virus was isolated from *A. robertsi*; Quaranfil virus and Gissar virus were identified in *A. vulgaris*; and “Issyk-Kul” virus has been identified in *C. vespertilionis*. No virus has been detected in ticks collected in China. The symptoms or diseases caused by these viruses are still unclear [11-13,16], and the vector roles of these ticks in China remain unknown. *Carios capensis* can be coinfected by pathogen DNA from *Borrelia*, *Coxiella*, and *Rickettsia*, as well as West Nile virus [14,15], although no pathogens have been reported in this tick species collected in China. *Ornithodoros tartakovskyi* and *O. tholozani* both cause tick-borne relapsing fever in China, but carry different pathogens, *B. latyshevyi* and *B. persica*, respectively [17].

**Table 1:** **Tick-borne pathogens and the vector role of argasid ticks distributed in China**

**Tick species**	**Pathogens**	**Diseases**	**References**
*Argas*			
*A. japonicas*	Unidentified	Dermatitis	[6]
*A. persicus*	*Borrelia anserine*; Kyasanur Forest disease virus^a^; *Wolbachia persica,* n. sp.^a^	Avian spirochetosis; Kyasanur forest disease; Paralysis	[7-10]
*A. robertsi*	Lake Clarendon virus^a^	unknown	[11]
*A. vulgaris*	Quaranfil virus^a^; Gissar virus^a^	unknown	[12,13]
*Carios*			
*C. capensis*	West Nile Virus^a,b^; *Borrelia*, *Coxiella*, and *Rickettsia* ^a,b^	West Nile fever	[14,15]
*C. vespertilionis*	“Issyk-Kul” virus^a^	unknown	[16]
*Orithodorous*			
*O. tartakovskyi*	*B. latyshevyi*	Tick-borne relapsing fever	[17]
*O. tholozani*	*B. persiea*	Tick-borne relapsing fever	[17]

^a^These pathogenic microorganisms have been recorded outside China.

^b^These pathogenic species have been detected within ticks or have been shown to be transmitted by ticks under controlled experimental conditions.

### Ixodid ticks in China, their roles as vectors, and associated tick-borne pathogens

There are 104 species of ixodid ticks in China in seven genera: *Amblyomma* (eight species), *Anomalohimalaya* (two species), *Dermacentor* (twelve species), *Haemaphysalis* (forty four species), *Hyalomma* (six species), *Ixodes* (twenty four species), and *Rhipicephalus* (eight species) [4]. Of these, 52 species from six genera have been shown to carry or transmit pathogenic microorganisms: *Ixodes* (seven species), *Amblyomma* (three species), *Dermacentor* (nine species), *Haemaphysalis* (twenty one species), *Hyalomma* (five species), and *Rhipicephalus* (seven species) (Table 2) [18-114]. Of these 52 species, 32 occur in China (Table 2). Tick-borne spotted fever is the most commonly detected disease, carried by at least 27 tick species. Lyme disease and human granulocytic anaplasmosis are the second and third most widespread tick-borne diseases, transmitted by at least 13 and 10 tick species, respectively (Table 2). Eight tick species are vectors for human granulocytic ehrlichiosis, seven tick species carry tick-borne encephalitis and babesiosis, and six species transmit hemorrhagic fever. The ixodid ticks that act as vectors of *Babesia* are usually coinfected with more than one *Babesia* species. These ticks include *I. persulcatus*, *D. nuttalli*, *Rh. microplus*, and *Rh. haemaphysaloides*, which are often infected by *Babesia bigemina* and *Ba. bovis* (Table 2).

**Table 2:** **Tick-borne pathogens and the role of ixodid ticks as vectors within China**

**Tick species**	**Pathogens**	**Diseases**	**References**
***Ixodes***			
*I. persulcatus*	*B. burgdorferi*; human granulocytc *Ehrlichia* (HGE)^b^; Spotted Fever Group *Rickettsia* (SFGR) ^b^; *Ehrlichia* ^b^; *Anaplasma phagocytophila* ^b^; tick-borne Encephalitis virus (TBEV); *Babesia bigemina*, *Ba. bovis*	Lyme disease; Ehrlichiosis; spotted fever; human granulocytic anaplasmosis; babesiosis	[18-25]
*I. kazakstani*	*B. burgdorferi* ^a^	Lyme disease	[26]
*I. nipponensis*	*B. afzelii* ^a,b^; TBEV ^a,b^	Lyme disease; tick-borne encephalitis;	[27,28]
*I. ovatus*	Ehrlichia ^a,b^; TBEV; *R. japonica* ^a,b^	Ehrlichiosis; tick-borne encephalitis; Oriental spotted fever	[29-31]
*I. granulates*	*B. burgdorferi*	Lyme disease	[32]
*I. acutitarsus*	*B. burgdorferi*	Lyme disease	[33]
*I. sinensis*	*R. monacensis* ^b^; *Ehrlichia, Bartonella, and Borrelia* ^b^; *B. garinii* ^b^	Lyme disease; Mediterranean Spotted Fever	[34]
***Amblyomma***			
*Am. geoemydae*	Reptile-associated *Borrelia spp.* ^a,b^;relapsing fever *Borrelia sp.* ^a,b^	Relapsing fever	[35]
*Am. helvolum*	SFGR ^a,b^; *Rickettsia* sp. ^a,b^	Spotted fever	[36,37]
*Am. testudinarium*	*R. tamurae* sp. nov.^a,b^; *Ehrlichia chaffeensis* ^b^;	Human monocytic ehrlichiosis	[38,39]
***Haemaphysalis***			
*H. longicornis*	New bunyavirus^b^; *B. burodorferi*; *A. phagocytophilum* ^b^; SFGR^a,b^; *Babesia* sp^a^; Huaiyangshan virus^b^; *Borrelia*, *Bartonella*, *Anaplasma*, and *Ehrlichia* ^b^; *Theileria uilenbergi* ^b^;	Severe fever with thrombocytopenia syndrome; Lyme disease; human granulocytic anaplasmosis; spotted fever; babesiosis; Huaiyangshan hemorrhagic fever	[40-47]
*H. concinna*	*B. garinii* ^b^; HGE^b^; SFGR^b^; TBEV^b^	Human granulocytic Ehrlichiosis	[23,48-50]
*H. punctata*	*B. burgdorferi* sensu stricto^b^; *Ba. major* and *T. orientalis*; Crimean-Congo hemorrhagic fever virus^a^; *Rickettsia* ^a,b^; *R. aeschlimannii* ^a,b^; *An. phagocytophilum* ^b^; *Flavivirus* ^b^	Lyme disease; Babesiosis; tick-borne encephalitis; Crimean-Congo hemorrhagic fever	[3,51-55]
*H. cornigera*	*R. heilongjiangensis* ^a,b^	Spotted fever	[31]
*H. erinacei*	SFGR^a,b^	Spotted fever	[56]
*H. flava*	*Ehrlichia* ^b^; *R. japonica* ^a,b^	EhIichiosis; Japanese Spotted fever	[57,58]
*H. formosensis*	*R. asiatica* sp. nov.^a,b^; Kyasanur Forest disease virus^a,b^; *R. japonica* ^a,b^; *An. phagocytophilum* ^a,b^	Spotted fever; Kyasanur Forest disease;	[31,59-61]
*H. hystricis*	*An. phagocytophilum* ^b^; *R. japonica* ^a,b^	Human granulocytic anaplasmosis; Japanese Spotted fever	[31,62]
*H. japonica*	*B. garinii* ^b^; TBEV^a,b^	Lyme disease; tick-borne encephalitis	[63,64]
*H. kitaokai*	SFGR^a,b^	Spotted Fever	[65]
*H. lagrangei*	*Anaplasma* spp.^a,b^; *Rickettsia* ^a,b^;	Human granulocytic ehrlichiosis; Rickettsioses	[66,67]
*H. bispinosa*	*B. burodorferi*; *T. sergenti*; *Ba. bigemina*	Lyme disease; Piroplasmosis	[32,68,69]
*H. megaspinosa*	*A. bovis* and *An. phagocytophilum* ^a,b^; SFGR^a,b^	Human granulocytic anaplasmosis; spotted fever;	[65,70]
*H. ornithophila*	SFGR^a,b^	Spotted Fever	[71]
*H. phasiana*	TBEV^a,b^	Tick-borne encephalitis	[64]
*H. qinghaiensis*	*T. uilenbergi* ^b^; *An. phagocytophilum* ^b^; *Theileria* spp.;	Human granulocytic anaplasmosis; theileriosis	[72-74]
*H. spinigera*	Flavivirus^a,b^	Kyasanur forest disease	[3]
*H. tibetensis*	GRD spirochetes	Unknown	[75]
*H. wellingtoni*	Kyasanur forest disease virus^a,b^; *Eubacterium* sp. strain Hw124 and *Eubacterium* sp. strain Hw191^a,b^	Kyasanur forest disease;	[66,76]
*H. campanulata*	*Coxiella burneti* ^b^	Q fever	[77]
*H. yeni*	*R. sibirica* ^b^	North-Asia Spotted fever	[78]
***Hyalomma***			
*Hy. anatolicum*	*Trypanosoma theileri*-like flagellates^a,b^; Crimean-Congo haemorrhagic fever virus^a,b^; *T. annulata*	Crimean-Congo haemorrhagic fever	[79-81]
*Hy. asiaticum*	Hemorrhagic fever virus^b^; *R. mongolotimonae* ^b^; *T. annulata*; *Rickettsiale* ^b^	Hemorrhagic fever; theileriosis	[81-84]
*Hy. scupense*	*T. annulata*	Theileriosis	[81]
*Hy. rufipes*	*T. annulata* ^a,b^	Theileriosis	[85]
*Hy. dromedarii*	Kadam virus^a,b^	Unknown	[86]
***Dermacentor***			
*D. nuttalli*	*B. burgdorferi* ^b^; SFGR^b^; HGE; *Ba. caballi* and *Ba. equi* ^a,b^; Rickettsiaes	Lyme disease; North Asia Spotted Fever; human granulocytic ehrlichiosis; babesiosis	[23,84,87-89]
*D. reticulatus*	*R. helvetica* ^a,b^; *R. slovaca* ^a,b^; *An. phagocytophila* ^a,b^; *Babesia* ^a,b^	Unexplained febrile illness; Spotless rickettsiosis; human granulocytic anaplasmosis; babesiosis	[90-93]
*D. silvarum*	HGE^b^; *E. chaffeensis* ^b^; TBEV; *R. raoultii* sp. nov.^b^; *R. heilongjiangensis* ^a,b^, *R. sibirica* ^b^	Human granulocytic ehrlichiosis; Encephalitis; Spotted Fever;	[23,94-98]
*D. auratus*	SFGR^b^	Spotted Fever	[99]
*D. everestianus*	*An. ovis* ^b^; *R. raoultii*–like bacteria^b^; *Bacillus tularensis* ^b^	Spotted Fever; Tularemia	[100-102]
*D. marginatus*	*B. burgdorferi* ^b^; *R. slovaca* ^a,b^	Lyme disease; tick-borne lymphadenopathy	[51,103]
*D. niveus*	SFGR^a,b^; *An. ovis* ^b^; *Bacillus tularensis* ^b^	Spotted Fever; Tularemia	[100,102,104]
*D. sinicus*	SFGR^b^	Spotted fever	[105]
*D. taiwanensis*	*R. japonica* ^a,b^;	Japanese Spotted fever	[106]
***Rhipicephalus***			
*Rh. microplus*	*A. marginale* ^b^, *Ba. bigemina*, *Ba. bovis*, *T. equi*; *E. chaffeensis* ^a,b^; TBEV^b^; *C. burnetii* ^b^	Babesiosis; theileriosis; Encephalitis; Q fever	[2,107,108]
*Rh. bursa*	*An. marginale* ^b^, *An. ovis* ^b^, *An. phagocytophilum* ^b^, *Ba. bigemina* ^b^, *Ba. ovis* ^b^, Bhanja virus^a,b^, Crimean-Congo hemorrhagic fever virus^a,b^	Human granulocytic anaplasmosis; babesiosis; Crimean-Congo hemorrhagic fever	[3,109]
*Rh. pumilio*	SFGR^a,b^; *R. conorii* ^b^; *An. phagocytophilum* ^b^	Spotted fever; human granulocytic anaplasmosis	[110,111]
*Rh. rossicus*	West Nile virus^a,b^	West Nile fever	[112]
*Rh. sanguineus*	*Ba. vogeli* ^b^, *E. canis* ^b^, *R. conorii* ^a,b^, *R. massiliae* ^a,b^, *R. rickettsii* ^a,b^	Babesiosis; Mediterranean spotted fever	[3,113]
*Rh. turanicus*	*R. conorii* ^a,b^, *R. massiliae* ^a,b^	Mediterranean spotted fever	[114]
*Rh. haemaphysaloides*	SFGR^b^; *Ba. bigemina* ^b^, *B. bovis* ^b^	Spotted fever; Babesiosis	[109]

^a^These pathogenic microorganisms have been recorded outside China.

^b^These pathogenic species have been detected within ticks or have been shown to be transmitted by ticks under controlled experimental conditions.

### Genus *Ixodes*

*Ixodes persulcatus* is undoubtedly the most notorious tick within China, and is known to carry a wide range of microorganisms, including *Borrelia*, *Ehrlichia*, *Rickettsia*, *Anaplasma*, and *Babesia* [18-25]. Lyme disease is mainly transmitted by *Ixodes* ticks, and *Borrelia* spp. have been isolated from or detected in *I. persulcatus*, *I. kazakstani*, *I. nipponensis*, *I. granulates*, *I. acutitarsus*, and *I. sinesis* in China [18,26,27,32-34]. Tick-borne encephalitis virus is carried by *I. persulcatus*, *I. nipponensis*, and *I. ovatus* [24,28,30], whereas spotted fever can only be transmitted by *I. persulcatus* and *I. ovatus* [20,31].

Among these *Ixodes* species, only *I. kazakstani* and *I. nipponensis* have not yet been shown to carry Lyme disease in China, because *B. burgdorferi* has not been found in *I. kazakstani* collected in China [26], and *B. afzelii* has not been detected in *I. nipponensis* distributed in China [28]. Although their pathogens have not been confirmed in China, the vector roles of these ticks are widely recognized [26,28]. Tick-borne encephalitis virus has not been found in *I. kazakstani* in China [27], whereas *Ehrlichia* and *R. japonica* have only been found in the species *I. ovatus*, distributed outside China [29,31].

### Genus *Amblyomma* (*Am*.)

*Amblyomma geoemydae* [35], *Am. helvolum* [36,37], and *Am. testudinarium* [38,39], collected from Japan, Thailand, and China, are known to carry pathogen DNA from *Borrelia*, *Rickettsia*, and *Ehrlichia*, respectively. However, although all these species are found in China, *E. chaffeensis*, detected in *Am. testudinarium*, is the only bacterial species that has been found in specimens collected within China [38]*.*

### Genus *Haemaphysalis*

The majority of ixodid ticks found in China belong to the genus *Haemaphysalis*. Globally, 21 of the 44 species found within China are known to be associated with pathogens. Of these 21 species, 11 (*H. longicornis*, *H. concinna*, *H. punctata*, *H. flava*, *H. hystricis*, *H. japonica*, *H. bispinosa*, *H. qinghaiensis*, *H. tibetensis*, *H. campanulata*, and *H. yeni*) have been confirmed as pathogen vectors in China (Table 2) [40-78]. The most commonly detected diseases vectored by this genus of ticks are spotted fever and human granulocytic anaplasmosis, which are transmitted by 11 and six species, respectively. *Borrelia* is carried by at least five species of this genus, and *Babesia* by at least four species (Table 2).

The ticks *H. longicornis*, *H. punctata*, and *H. concinna* support the greatest diversity of pathogenic microorganisms, with *H. longicornis* the major vector of *B. burgdorferi*, *Theileria* spp., *Coxiella burnetti, Babesia* spp., *Anaplasma phagocytophilum*, *Ehrlichia*, *Bartonella*, spotted-fever-group rickettsiae, Huaiyangshan virus, and the recently identified New bunyavirus (Table 2), which has caused many deaths in China, Japan, and Korea [40-47]. *Haemaphysalis concinna* is mainly distributed in northern China, where multiple outbreaks of *H. concinna*-borne disease have been reported since the early 20th century. These outbreaks have been attributed to a diverse array of pathogens, including *B. garinii*, human granulocytic *Ehrlichia*, spotted-fever-group Rickettsiae, and encephalitis viruses [23,48-50]. *Haemaphysalis punctata* transmits *B. burgdorferi sensu stricto*, *Ba. major*, *T. orientalis*, Crimean–Congo hemorrhagic fever virus, *Rickettsia*, *R. aeschlimannii*, *An. phagocytophilum*, and *Flavivirus*, resulting in diseases such as Lyme disease, babesiosis, tick-borne encephalitis, and Crimean-Congo hemorrhagic fever [3,51-55]. *Haemaphysalis formosensis* has been shown to carry pathogen DNA from a number of bacterial species, including *R. asiatica* sp. nov., Kyasanur Forest disease virus, *R. japonica*, and *An. phagocytophilum*, but these pathogens have not yet been detected in this tick species within China. Among the pathogenic microorganisms transmitted by *Haemaphysalis* species, most have been characterized with molecular techniques, and some species have been shown to transmit particular pathogens under controlled experimental conditions (Table 2).

### Genus *Hyalomma* (*Hy*.)

Five species of *Hyalomma* are known to harbor pathogenic microorganisms (Table 2) [79-86], and three have been confirmed as vectors within China: *Hy. anatolicum*, *Hy. asiaticum*, and *Hy. scupense. Hyalomma anatolicum* and *Hy. asiaticum* carry the greatest diversity of pathogens, and each transmits at least three pathogens. *Theileria annulata* is the most common pathogenic microorganism, and is transmitted by four of the five *Hyalomma* vector ticks (*Hy. anatolicum*, *Hy. asiaticum*, *Hy. scupense*, and *Hy. rufipes*) [79,85]. *Trypanosoma theileri*-like flagellates and Crimean-Congo hemorrhagic fever virus have been detected in *Hy. anatolicum* outside China, whereas *T. annulata* was characterized from *Hy. anatolicum* within China [79-81]. Hemorrhagic fever virus and *R. mongolotimonae* were detected in *Hy. asiaticum* in north China [81-83], and *Hy. asiaticum* is the only tick species that can transmit Rickettsiae [84]. *Hyalomma dromedarii* has been shown to transmit Kadam virus outside China, although the resulting symptoms are still unknown [86].

### Genus *Dermacentor*

Nine of the 12 species of *Dermacentor* found within China can transmit pathogens, and seven of these species (*D. nuttali*, *D. silvarum*, *D. auratus*, *D. everestianus*, *D. marginatus*, *D. niveus*, and *D. sinicus*) are of epidemiological importance in China (Table 2) [23,84-106]. The widely distributed *D. nuttalli*, *D. reticulates*, and *D. silvarum* carry the largest numbers of different pathogenic microorganisms, and Rickettsiae are the most commonly found bacteria in this genus (Table 2). The causative agent of human granulocytic ehrlichia has been detected in *D. silvarum* and *D. nuttalli* within China [23], and *Babesia* is commonly found in *D. nuttalli* and *D. reticulatus* outside China [89]. *Borrelia burgdorferi* has been found in *D. marginatus* [51] and *D. nuttalli* within China [87]; *An. ovis* and *Bacillus tularensis* are most commonly found in *D. everestianus* [100] and *D. niveus* within China [104]; and *An. phagocytophila* is specifically detected in *D. reticulatus* outside China [92].

### Genus *Rhipicephalus* (*Rh*.)

In the genus *Rhipicephalus*, seven tick species are known to harbor pathogenic microorganisms, and five of these species (*Rh. microplus*, *Rh. bursa*, *Rh. pumilio*, *Rh. sanguineus*, and *Rh. haemaphysaloides*) are confirmed vectors in China (Table 2) [107-114]. *Rhipicephalus microplus* and *Rh. bursa* carry the largest numbers of different pathogens in this genus. *Ehrlichia chaffeensis*, Bhanja virus, and Crimean–Congo hemorrhagic fever virus have not yet been detected in *Rh. microplus* or *Rh. bursa* within China. *Babesia* is the most common microorganism transmitted by *Rh. microplus* [2], *Rh. bursa*, *Rh. sanguineus* [3], and *Rh. haemaphysaloides* [109], and various *Rickettsia* species have been found in *Rh. pumilio* [110], *Rh. sanguineus*, *Rh. haemaphysaloides*, and *Rh. turanicus*. West Nile virus and Crimean–Congo hemorrhagic fever virus have been characterized solely in *Rh. rossicus* [112] and *Rh. bursa* [109], respectively, whereas *Ehrlichia* has been found in both *Rh. microplus* [107] and *Rh. sanguineus* [113]. *Anaplasma marginale*, *An. ovis*, and *An. phagocytophilum* can be acquired by *Rh. bursa* [3,113]. Among these bacterial species, no spotted-fever-group *Rickettsia* has been detected in *Rh. pumilio* collected in China; West Nile virus has not been characterized in *Rh. rossicus* within China; *R. conori*, *R. massiliae*, and *R. rickettsii* have not been detected in *Rh. sanguineus* in China; and *R. massiliae* has not been detected in *Rh. turanicus* within China.

## Conclusion

Of the estimated 117 species of ticks in China, 36 have been confirmed to carry or transmit one or more pathogens, and 24 additional species are known to be pathogenic vectors in other countries. Furthermore, 38 species have been shown to carry multiple pathogens, indicating the major roles they play in the spread and transmission of these pathogens. Therefore, the number of pathogens and the vector potential of ticks may still be underestimated, because of the complex distributions and the great diversity of tick species in diverse ecological habitats in China. However, such knowledge will provide clues to the further identification of tick-associated pathogens, especially in epidemic areas with multiple tick species. Much more work is required to better distinguish between ticks that carry potential pathogens and those that are competent to transmit pathogens to a host. Targeted prevention methods will then be more effective in controlling tick-borne diseases.
